# How hypoxia affects microbiota metabolism in mice

**DOI:** 10.3389/fmicb.2023.1244519

**Published:** 2023-09-28

**Authors:** Ainiwaer Ailizire, Xiaojing Wang, Yan Ma, Xin Yan, Shiqi Li, Ziyi Wu, Wenqi Du

**Affiliations:** ^1^Department of Public Health, Qinghai University School of Medicine, Xining, China; ^2^Department of Proctology, Qinghai Provincial Traditional Chinese Medicine Hospital, Xining, China; ^3^Research Center for High Altitude Medicine, Qinghai University School of Medicine, Xining, China; ^4^Key Laboratory for Application of High Altitude Medicine in Qinghai Province, Qinghai University, Xining, China

**Keywords:** hypoxia, mice, gut microbiota, metabolomics, correlative analyses

## Abstract

**Objective:**

To investigate the relationship between gut microbiota and the fecal metabolites of hypoxic environments in mice.

**Methods:**

High-fat diet-induced obese mice (*n* = 20) and normal diet-fed mice (*n* = 20) were randomly divided into four groups: high altitude obese group (HOB), high altitude normal weight group (HN), low altitude obese group LOB (LOB), and low altitude normal weight group (LN). Fecal samples from each group were 16S rRNA gene sequenced, and five samples from each of the four groups above were selected for non-targeted fecal metabolomics analysis using liquid chromatography-mass spectrometry. The relationship between gut microbiota and fecal metabolites was analyzed using SIMCA 14.1, MetaboAnalyst 5.0 and R 4.1.11.

**Results:**

(A) Body weight was significantly lower in the hypoxic obesity group than in the normoxic obesity group. (B) Differences in α-diversity and β-diversity were found in the fecal gut microbiota of mice of different body weights and altitude, and the diversity of gut microbiota was higher in the normal group than in the obese group; the results of the comparison between the two groups showed that Faecalibaculum, Romboutsia, Lactobacillus, and A2 were associated with obesity; Romboutsia was associated with hypoxia. (C) The metabolic profiles of fecal metabolites differed between groups: gut microbiota were associated with nucleotide and amino acid metabolism in the same body groups, while gut microbiota were associated with lipid and amino acid metabolism in the same oxygen concentration groups.

**Conclusion:**

(a) Gut microbiota diversity was reduced in obese groups. Romboutsia was the dominant microbiota in the hypoxia group. (b) Gut microbiota were associated with nucleotide and amino acid metabolism in the same body weight groups, while they were associated with lipid and amino acid metabolism in the same altitude groups.

## Introduction

Recently, obesity has become a major public health issue of worldwide concern (Nam et al., 2015). Obesity is associated with several metabolic diseases, such as type 2 diabetes, cardiovascular diseases, metabolic syndrome, and nonalcoholic fatty liver disease, which threaten both physical and mental health (Xie et al., 2010; Ding et al., 2015; Pérez-Pérez et al., 2017). The etiology and mechanisms of obesity are extremely complex, involving interactions between genetic and environmental factors. Among the nongenetic factors associated with obesity, intestinal flora composition has been recognized as a regulator of obesity, and a correlation between changes in the intestinal flora composition and body weight has been observed in a number of studies conducted in animal models of obesity (Ley et al., 2006; Jumpertz et al., 2011).

The dynamic balance of the gut microbiota is one of the most important indicators for maintaining human health, and it has been proposed that the composition and metabolic functions of the gut microbiota may influence the development of obesity (Torres-Fuentes et al., 2017; Abenavoli et al., 2019; Aron-Wisnewsky et al., 2020). The gut microbiota has been recognized as an important factor in the development of metabolic diseases such as obesity, and is considered an endocrine organ involved in the maintenance of energy homeostasis and host immunity (Gomes et al., 2018). The hypoxic environment is associated with changes in the host gut microbiota and the production of many metabolites that affect normal physiological levels in the host (Pral Laís et al., 2021). A study of gut microbial changes in mice exposed to different concentrations of oxygen found that the diversity of the gut flora was altered in mice exposed to hypoxia and that the metabolism of the mice was altered (Zhang et al., 2018). Zhang et al. (2018) used mice as an animal model to analyze the fecal microbiota of mice fed for 30 days at different altitudes with different oxygen levels, and studied the influence of high altitude and low oxygen environments on the gut microbiota, the composition and flora of the gut microbiota of mice fed at different altitudes β Significant differences in diversity occurred. In a study of dynamic changes in the gut microbiota of rats exposed to hypoxia (50 KPa, 380 mmHg), metagenomic sequencing was performed on faces from SD rats entering the hypoxic chamber (8 time points) and exiting the hypoxic chamber (6 time points) at 14 time points. The results showed α- diversity changes within the first 5 days of entering or leaving the hypoxic chamber, while β-diversity analysis showed that the structure of the gut microbiota was clearly separated at 14 time points. After entering the hyperbaric oxygen chamber, the relative abundance of bacteria decreased. The most abundant gut microbiota at the genus level was *Prevotella* (Han et al., 2022). Therefore, we believe that there is a strong correlation between individual location and differences in gut microbiota.

Metabolomics, as an emerging research tool and method in the post genomic era, is one of the most used phenotypic research methods and has become an indispensable research tool in science (Nicholson et al., 1999; Schoeman et al., 2016). It reflects changes at the most terminal level of various biochemical processes in the human body, such as various vital activities and disturbances of metabolic homeostasis caused by disease and drug stimulation, and is also influenced by factors such as the living environment and lifestyle, such as differences in dietary habits, air quality, and frequency and intensity of physical activity (Wishart et al., 2007, 2018). Fecal metabolites may reflect the status of the gut microbiota and the relationship between the symbiotic flora and the host. The combination of fecal metabolomics and 16S rRNA gene sequencing can help explain the close relationship between the gut microbiota and the host (Zhou et al., 2020). Researchers found a comprehensive landscape of gut microbiota and metabolite profiles in patients with SCI, providing evidence that their interaction plays a role in the pathogenesis of SCI, and findings suggest that uridine, hypoxanthine, PC(18:2/0:0) and kojic acid may be important therapeutic targets for the treatment of this condition (Kong et al., 2023). In a study of the effects of semaglutide on skeletal muscle and its metabolomics, it was found to significantly reduce body weight and intramuscular fat accumulation in obese mice, possibly by altering the metabolism of muscle lipids and organic acids (Ren et al., 2022). In this study, *Akkermansia* abundance was found to be positively correlated with levels of secondary bile acids, and secondary bile acids restore and rebuild a healthy microbiome as a means to intervene in aging (Bárcena et al., 2019). Untargeted metabolomics and 16S rRNA gene sequencing revealed that *Lachnoclostridium, Fusobacterium, Coprococcus_2*, and *Tyzzerela* are correlated with arachidonic acid, taurocholic acid, and DHEA sulfate, which can be used as characteristic metabolites for obese patients (Zhou et al., 2020). Metabolic association analysis of gut microbiota could be used to find therapeutic targets for various diseases.

So, in this study, we used a high-fat diet to construct an obese mouse model through hypoxia intervention, combined with 16S rRNA gene sequencing technology and a non-targeted targeted metabolomics approach to reveal the effect of hypoxia on flora and metabolic changes, and further reveal its mechanism on the occurrence of obesity.

## Materials and methods

### Ethics statement

Animal care and experimental treatments were approved by the Medical Science Research Ethics Committee of Qinghai University (No. 2021-06, approved 21 June 2021).

### Animals and experimental design

A total of 64 male C57BL/6 mice, aged 5 weeks and weighing 16–17 g, were purchased from Hunan Slake Jingda Experimental Animal Co., Ltd. Mice were raised in an environment with a temperature of 18–23°C, and provided with sufficient food and water. After 1 week of adaptive feeding, the animals were randomly divided into a normal control group (NC, *n* = 24) and high-fat diet group (HFD, *n* = 40). HFD groups were fed a high-fat diet, while the NC group were fed with a normal diet. The feed source came from D12492, Beijing Keao Xieli Feed Co., Ltd. The feed inclued Casein, L-cystine, Corn Starch, Maltodextrin 10, Sucrose, Celluse, Soybean Oil, Lard, Mineral Mix S10026^*^, Dicalcium phosphate, Calcium Carbonate, Potassium Citrate (1 H2O), Vitamin Mix, V10001, Choline Bitartrate, and FD&C Blue Dye # 1. The NC group was fed a standard diet (3.18 kcal/g, 4.0 g% fat, 67.3% carbohydrate, 19.1% protein). The HFD was fed a high-fat diet (5.24 kcal/g, 34.9% fat, 26.3% carbohydrate, 26.2% protein). After 8 weeks (13 weeks of age), the mice were fasted for 12 h and weighed, and the weight of the HFD group exceeded the average weight of the NC group by 10% as the criterion for judging the successful modeling of obese mice. After successful modeling, 20 male C57BL/6J obese mice and 20 normal feed mice were randomly divided into four groups: high altitude obese group (HOB), high altitude normal weight group (HN), low altitude obese group LOB (LOB), and low altitude normal weight group (LN). HOB and LOB groups were placed in a low-pressure oxygen chamber (simulated altitude of 5,000 m, atmospheric pressure of 425 mmHg, oxygen partial pressure of 72.5 mmHg, and oxygen concentration of 13.83%), LOB and LN groups were kept in a laboratory environment (altitude of 2,261 m, mean atmospheric pressure of 574 mmHg, oxygen partial pressure of 120.1 mmHg, and oxygen concentration of 17.5%) for 4 weeks. Body weight and food consumption were measured weekly; fecal gut microbiota and fecal metabolomics analysis were measured after 30 days.

### Collection of fecal samples

Sterile plastic cassettes were used to collect fresh stool samples (5 g) from each group. The fecal samples were quickly placed in an ice box, transported to the laboratory with 2 h, and then stored at −80 in a refrigerator for further processing and testing.

### 16s rRNA gene sequencing

Total genomic DNA was extracted from the samples using the acetyltrimethylammonium bromide (CTAB) method (Attitalla, 2011). DNA concentration and purity were analyzed using 1% agarose gels. According to the concentration, the DNA samples were diluted to 1 ng/μl using sterile water. An equal volume of 1 × loading buffer (containing SYBR green) was mixed with the PCR products, and electrophoresis was performed on a 2% agarose gel to visualize the PCR products. PCR products were mixed in equidensity ratios. Then, the PCR products were purified with a Qiagen Gel Extraction Kit (Qiagen, Germany). The DNA purity and concentration were analyzed by measuring the optical density (OD) at wavelengths of 260 and 280 nm with a NanoPhotometer R spectrophotometer (Implen, Munich, Germany) and then calculating the OD260:OD280 ratio. The DNA concentrations were measured with the Qubit R dsDNA Assay Kit in a Qubit R 2.0 Fluorometer (Life Technologies, Camarillo, CA, United States) (Du et al., 2022).

Based on many previous studies (Nagayama et al., 2020; Yu et al., 2020; Song et al., 2021), we selected the V3–V4 region to study the microbiome through second-generation sequencing. 16S rRNA gene sequencing was performed by Novogene Bioinformatics Technology Co., Ltd., China. DNA samples were diluted to a concentration of 1 ng/μl in sterile water and then PCR amplified with the 515F/806R primer set (515F: 5′GTGCCAGCMGCCGCGGTAA-3′, 806R: 5′-XXXXXXGG ACTACHVGGGTATCTAAT-3′). Sequencing libraries were generated using the TruSeq R DNA PCR-Free Sample Preparation Kit (Illumina, USA) following the manufacturer's recommendations, and index codes were added. Library quality was assessed with a Qubit@ 2.0 Fluorometer (Thermo Scientific) and Agilent Bioanalyzer 2100 system. Finally, the library was sequenced on the Illumina NovaSeq platform, and 250-bp paired-end reads were generated.

Paired-end reads were assigned to samples based on their unique barcodes and were truncated by removing the barcode and primer sequences. Paired-end reads were merged using FLASH (V1.2.7) (Magoč and Salzberg, 2011), which is a very fast and accurate analysis tool that was designed to merge paired-end reads when at least some of the reads overlapped with the read generated at the opposite end of the same DNA fragment. The splicing sequences are called raw tags. Quality filtering of raw tags was performed under specific filtering conditions to obtain high-quality cleantags (Bokulich et al., 2013) according to the QIIME (V1.9.1) (Caporaso et al., 2010) quality control process. The tags were compared with those in the reference database (Silva138 database) using the UCHIME (Attitalla, 2011) algorithm to detect chimeric sequences, and the chimeric sequences were removed (Haas et al., 2011). Then, effective tags were finally obtained.

The Kruskal–Wallis test was used to investigate significant differences in operational taxonomic units (OTUs) and the abundance-based coverage estimator (ACE), Chao 1, Simpson's, and Shannon's indexes among the four groups. To correct for type I errors, we applied the Bonferroni method for multiple comparisons between two groups. Differences in microbial community abundances between the obesity group and the control group were analyzed using the Wilcoxon rank sum test, and the significance of these differences was assessed using the false discovery rate (FDR). Principal coordinate analysis (PCoA) was performed using the WGCNA package, stat packages, and ggplot2 package in R software (v 2.15.3) to compare the similarity of community structures. Multiresponse permutation procedures (MRPPs) (O'Reilly and Mielke, 1980) were used to analyze differences in the microbial community structure between groups.

### Analysis of untargeted metabolomics

The supernatant of the stool sample was taken after grinding and centrifugation. The QC samples were prepared by mixing the supernatants of all samples in equal quantities. UHPLC-MS/MS analyses were performed using a Vanquish UHPLC system (ThermoFisher, Germany) coupled with an Orbitrap Q Exactive TM HF mass spectrometer (Thermo Fisher, Germany). Samples were injected onto a Hypesil Goldcolumn (100 × 2.1 mm, 1.9 μm) using a17 min linear gradient at a flow rate of 0.2 mL/min. The eluents for the positive polarity mode were eluent A (0.1% FA in Water) and eluent B (Methanol). The eluents for the negative polarity mode were eluent A (5 mM ammonium acetate, pH9.0) and eluent B (Methanol). The solvent gradient was set as follows: 2% B, 1.5 min; 2–85% B, 3 min; 85–100% B, 10 min; 100–2% B, 10.1 min; 2% B, 12 min. Q Exactive TM HF mass spectrometer was operated in positive/negative polarity mode with spray voltage of 3.5 kV, capillary temperature of 320°C, sheath gas flow rate of 35 psi and aux gas flow rate of 10 L/min, S-lens RF level of 60, and Aux gas heater temperature of 350°C.

The raw data files generated by UHPLC-MS/MS were processed using the Compound Discoverer 3.1 (CD3.1, ThermoFisher) to perform peak alignment, peak picking, and quantitation for each metabolite, and these metabolites were annotated using the KEGG database, HMDB database, and LIPIDMaps database. Positive and negative data were combined to obtain a combined data set, which was imported into the SIMCA-P+14.0 software. Principal component analysis (PCA) was first used to observe the overall distribution of each sample and the stability of the whole analysis process. Then, OPLS-DA were used to distinguish the overall differences of metabolic profiles between groups and screen differential metabolites between groups. The differentially expressed metabolites should meet the importance of the first principal component variable in the OPLS-DA model at projection VIP > 1 and *P* < 0.05. Volcano plot (FC > 1.2 or FC < 0.833), *P* used Benjamin and Hochberg to check (FDR). Metabolic pathway analysis was performed using MetaboAnalyst 5.0 (https://www.metaboanalyst.ca/) software and for mapping. The data were subjected to multivariate (PCA) and orthogonal OPLS-DA and univariate [fold change (FC) and T-tests] analysis. Mean metabolite concentrations in each group were used to calculate FC values, Final identification of differential metabolites by VIP, FC and *P*.

### Statistical analysis

Quantitative data with normal distribution between the two groups were expressed by mean±standard deviation and analyzed by student *t* test. The quantitation sequencing data with non-normal distribution were expressed by median ± quartile range (QR) and analyzed by Wilcoxon rank sum test. Association analysis of gut microbiota and metabolites was carried out using spearman correlation. The SPSS 22.0 version was used for statistical analysis. *P* < 0.05 was considered as statistically significant different.

## Results

### Construction of a mouse obesity model

The results showed that there was no significant difference in body weight between the two groups at baseline (*P* > 0.05) after 8 weeks of feeding, and the body weight gain value of the high-fat diet group was higher than that of the normal control group in the same feeding environment. When the weight of the two groups was compared at the same time, there was a significant statistical difference in the weight of the two groups at each time point (*P* < 0.05) (Table 1).

**Table 1:** Changes in body weight of obese mice (g) ($\bar{x}$ ± s).

**Time**	**NC (*n* = 24)**	**HFD (*n* = 40)**	**Gain weight**	**t**	***P***
Week 0	16.07 ± 0.64	16.33 ± 0.64	0.26 ± 1.24	0.872	0.393
Week 1	18.06 ± 0.83	19.72 ± 0.89	1.66 ± 1.78	4.727	0.000
Week 2	19.68 ± 1.14	21.42 ± 0.86	1.75 ± 1.01	4.250	0.000
Week 3	20.79 ± 1.37	22.78 ± 1.07	1.99 ± 1.05	3.951	0.001
Week 4	21.46 ± 1.27	23.43 ± 1.04	1.97 ± 1.13	4.144	0.000
Week 5	22.57 ± 1.42	24.43 ± 1.02	1.86 ± 1.16	3.673	0.001
Week 6	23.46 ± 1.48	24.94 ± 1.09	1.48 ± 1.01	2.791	0.011
Week 7	22.12 ± 1.60	25.59 ± 1.17	3.47 ± 0.94	6.055	0.000
Week 8	21.64 ± 1.87	26.70 ± 1.48	5.06 ± 1.41	7.333	0.000

### Weight change in four groups of mice under hypoxic treatment

Twenty mice were randomly selected from the two groups with a high-fat diet (LOB and HOB), as were 20 mice from the normal diet groups. the weight change of mice after 4 weeks was shown in Table 2; the results showed that at the same altitude, there were statistical differences in body weight between the two groups between the normoxic group (2,261 m) and the hypoxic group (5,000 m) (*P* < 0.05). The comparison between the obese groups at different altitudes showed that the weight gain of the HOB was lower than that of the LOB, and the difference between the two groups was statistically significant (*P* < 0.05).

**Table 2:** Body weight changes of low hypoxia mice in four groups(g) ($\bar{x}$ ± s).

**Time**	**2,261 m**		**t**	***P***	**5,000 m**		**t**	***P***
	**LN (*****n*** = **10)**	**LOB (*****n*** = **10)**			**HN (*****n*** = **10)**	**HOB (*****n*** = **10)**		
Week 1	22.66 ± 0.66	28.53 ± 1.87	7.252	0.000	2.94 ± 0.29	26.16 ± 1.27^*^	4.072	0.000
Week 2	22.73 ± 0.91	28.46 ± 1.72	7.204	0.000	22.13 ± 1.86	26.12 ± 1.16^*^	5.756	0.000
Week 3	23.13 ± 0.96	28.92 ± 1.60	7.624	0.000	22.65 ± 1.92	25.60 ± 1.50^*^	2.531	0.021
Week 4	23.08 ± 0.87	28.96 ± 1.78	7.265	0.000	22.79 ± 1.69	26.23 ± 1.40^*^	3.516	0.003

* Compared with LOB group (*P* < 0.05).

### General characteristics of the four groups of mice

Mice heights were not different between altitudes and body weights (*P* > 0.05), and mice weights were statistically different between groups at the same altitude (*P* < 0.05). The body weight of the normoxic group was higher than that of the hypoxic group (Table 3).

**Table 3:** Basic characteristics of mice ($\bar{x}$ ± s).

**Index**	**2,261 m**		**t**	***P***	**5,000 m**		**t**	***P***
	**LN (*****n*** = **10)**	**LOB (*****n*** = **10)**			**HN (*****n*** = **10)**	**HOB (*****n*** = **10)**		
Hight (cm)	10.38 ± 0.13	10.13 ± 0.38	1.511	0.162	10.50 ± 0.55	10.37 ± 0.39	0.619	0.551
Weight (g)	24.12 ± 0.76	29.08 ± 0.85	13.75	0.000	22.51 ± 0.83^a^	26.33 ± 0.51^b^	12.400	0.000

a Compared with LN group (*P* < 0.05);

b Compared with HN group (*P* < 0.05).

### *α* and β diversity analysis of four groups of gut microbiota

**Figure 1:**
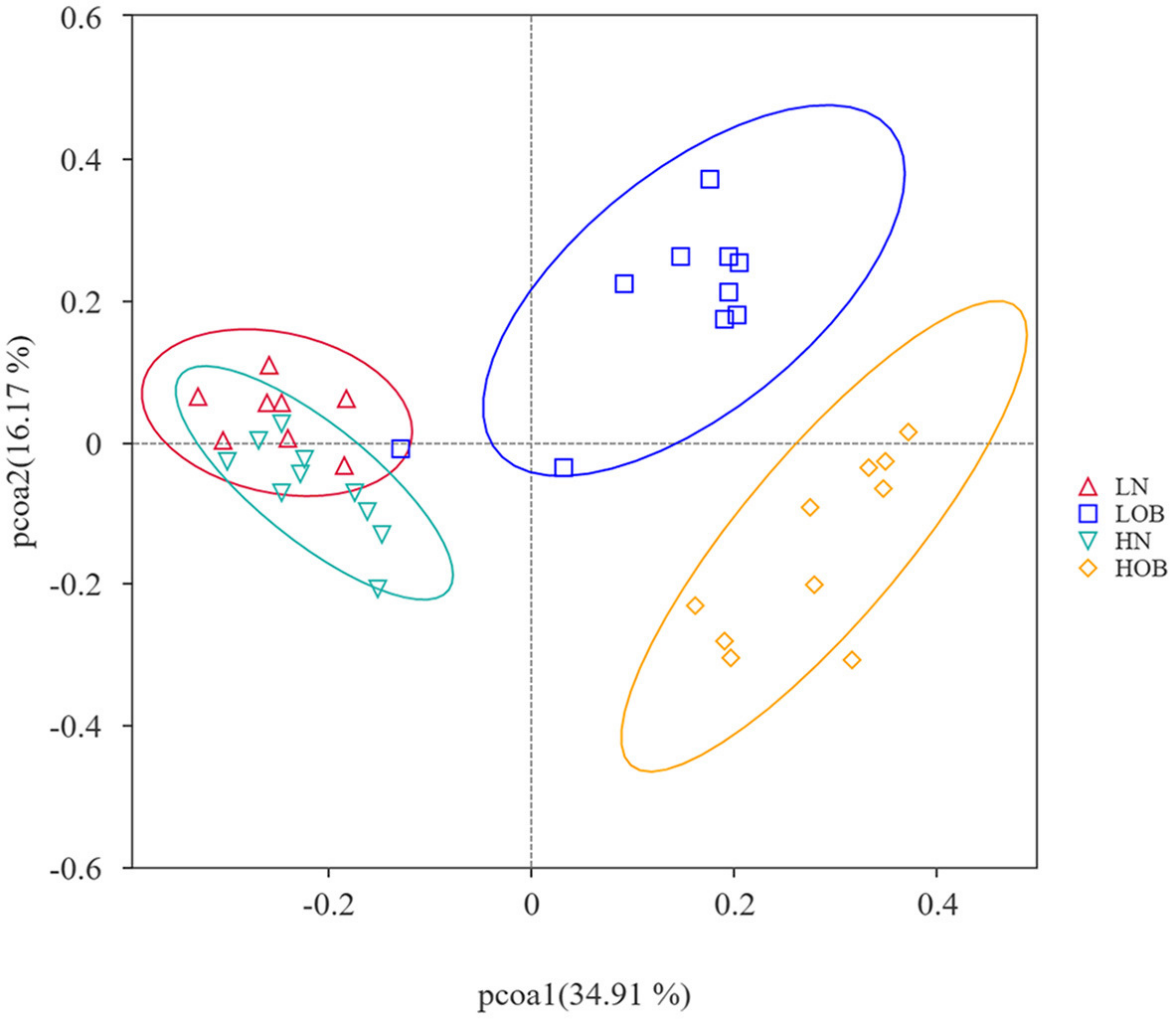
Beta diversity analysis based on UniFrac analysis. Orange dots represents the hyposia obese mice (HOB) group. Green dots represents the hyposia normal weight mice (HN) group. Blue dots represents the normoxic obese mice (LOB) group. Red dots represents the normoxic weight children (LN) group. Circles in orange, green, blue, and red represent different periodontal bacterial community clusters, respectively.

*α*-diversity analysis by Shannon, Simpson, Choa1, and ACE index showed that there were differences in α-diversity between the groups (*P* < 0.05). Further intergroup comparisons showed that in the same oxygen groups, Shannon, Simpson, Chao1, and ACE indices were significantly different in the HOB and HN groups (*P* < 0.05). Simpson index was significantly different in the LOB and LN groups (*P* < 0.05), suggesting that gut microbiota diversity was lower in the obese group than in the control group. In the same body weight groups, Chao1 and ACE indices were significantly different in HOB and LOB groups (*P* < 0.05, Table 4), suggesting that gut microbiota diversity was lower in the hypoxic obese group compared to the control group. All these results suggested that hypoxia and obesity affect the diversity of the gut microbiota. PcoA was used to analyze the β-diversity of fecal samples among the four groups, and the results showed that the gut microbiota composition of normal weight mice was similar; the gut microbiota composition of obese mice was significantly different at different oxygen levels (*P* < 0.05, Figure 1). Using MRPPs based on Bray-Curtis distance, there was a small difference between samples within each group and a large difference between groups (*P* < 0.05, Table 5).

**Table 4:** Alpha-diversity analysis of faces in mice.

**Group**	**Shannon**	**Simpson**	**Chao1**	**ACE**
LN	5.334 ± 0.698	0.913 ± 0.698	486.515 ± 39.647	492.314 ± 39.024
LOB	4.843 ± 0.841	0.857 ± 0.720^a^	499.291 ± 48.923	505.821 ± 49.218
HN	5.907 ± 0.257	0.957 ± 0.009	517.493 ± 29.088^a^	522.998 ± 26.675
HOB	4.931 ± 0.742^b^	0.890 ± 0.484^b^	452.293 ± 60.283^b^^c^	457.891 ± 60.393^b^^c^
F	0.005	0.001	0.025	0.025
*P*	0.011	0.007	0.054	0.054

a Compared with LN group (*P* < 0.05);

b Compared with HN group (*P* < 0.05);

c Compared with LOB group (*P* < 0.05).

**Table 5:** MRPP analysis of differences in microbial community structure between groups.

**Group**	**A**	**Observed delta**	**Expected delta**	***P***
LOB-LN	0.232	0.369	0.481	0.001
HOB-HN	0.228	0.428	0.554	0.001
HN-LN	0.101	0.389	0.432	0.001
HOB-LOB	0.163	0.411	0.491	0.001

Multiresponse permutation procedures (MRPP) analysis of differences in microbial community structure between group, A = 1 – (observed-delta/expected-delta), the smaller the Observe Delta value, the smaller the difference within the group, and the larger the Expect delta value, the larger the difference between the groups. A > 0, indicates that the difference between groups is greater than the difference within the group, A < 0, indicates that the difference within the group is greater than the difference between the groups.

### Gut microbiota composition among the four groups

**Figure 2:**
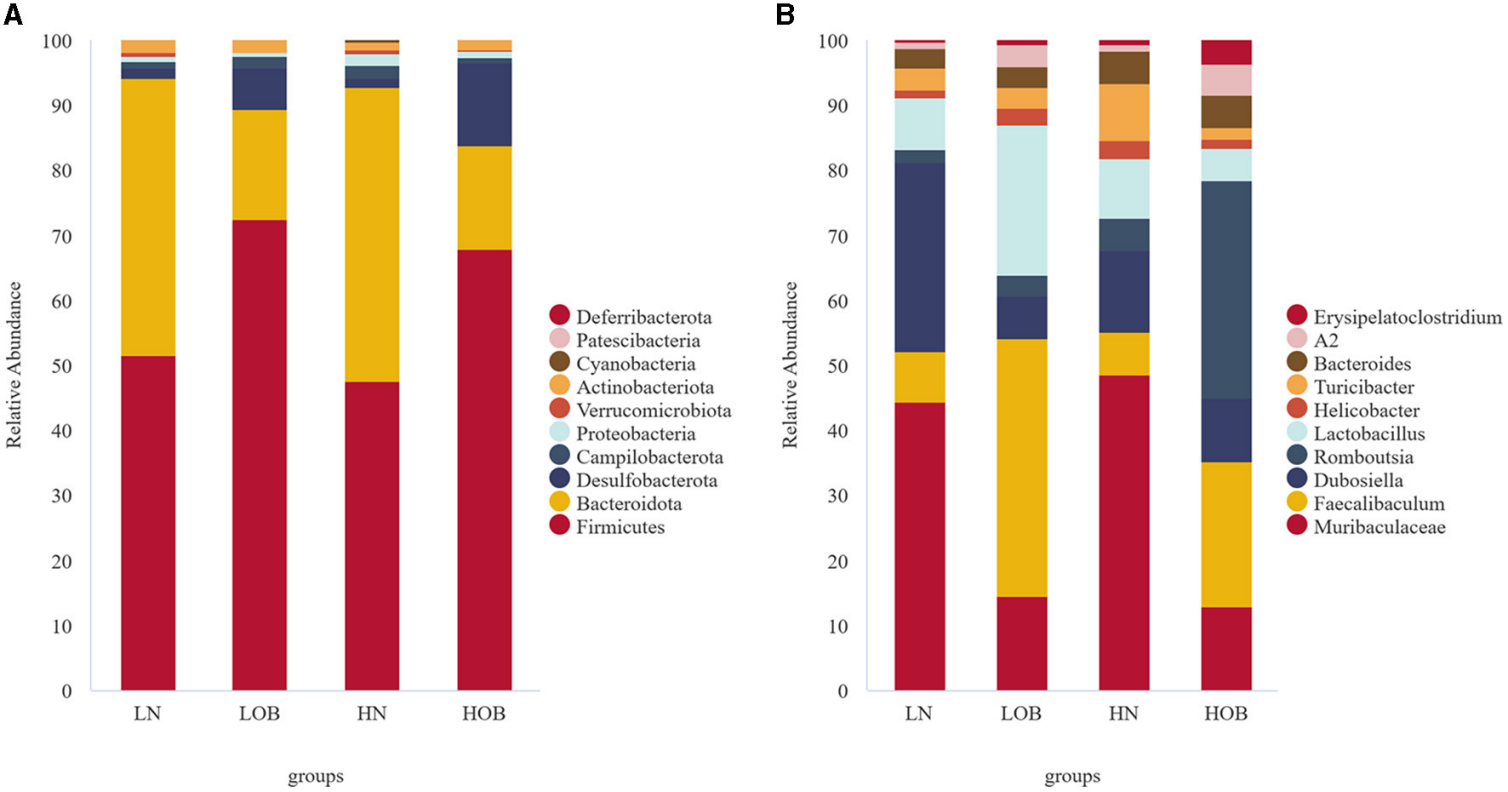
In phylum and genus level gut microbiota composition. **(A)** The phylum level. **(B)** The genus level.

Overall, 25 phylum, 38 classes, 92 orders, 139 families, 230 genera, and 117 species were detected in the bacterial microbiome communities of the four group samples. Composition at the phylum level: In the obese group, *Firmicutes, Bacteroidota*, and *Desulfobacterota* dominated, and their proportions were HOB group (67.96%, 15.85%, 12.68%) and LOB group (72.38%, 17.09%, 6.34%). Firmicutes/Bacteroidota in the HOB group was higher than in the LOB group. Normal body groups were dominated by *Firmicutes* and *Bacteroidota*, and their proportions were HN (47.61%, 45.14%) and LN (51.52%, 42.70%) (Figure 2A). At the genus level, the LN group was dominated by *Muribaculacea* (35.02%), *Faecalibaculum* (6.13%), and *Dubosiell* (22.90%), while the LOB group was dominated by *Muribaculaceae* (10.26%), *Faecalibaculum* (28.17%), and *Lactobacillus* (16.44%). The HN group was dominated by *Muribaculaceae* (34.82%) and *Dubosiella* (9.04%) and the HOB group by *Romboutsia* (21.01%) and *Faecalibaculum* (14.07%). Among them, the hypoxic obesity group was significantly dominated by *Romboutsia* (Figure 2B).

### Analysis of discrepancy among four groups

The Metastat method was used to analyze the differences in microbiota abundance composition of the top 10 microbiota at the genus level between the groups. For the same body weight groups, the HN vs. LN groups demonstrated that *Dubosiella, Romboutsia*, and *Turicibacter* were significantly different (*P*<0.05), and the abundance of *Romboutsia* and *Turicibacter* was higher in the HN group. In the HOB vs. LOB group, *Faecalibaculum, Romboutsia, Lactobacillus, Turicibacter*, and *Erysipelatoclostridium* were significantly different (*P*<0.05), the abundance of *Romboutsia* was higher in the HOB group, and *Romboutsia* was significantly dominant in the hypoxia group. At the same oxygen level, in the LOB and LN groups, *Muribaculaceae, Faecalibaculum, Dubosiella, Lactobacillus*, and *A2* showed significant differences (*P*<0.05), and *Faecalibaculum* and *Lactobacillus* had higher abundance in LOB. In the HOB and HN groups, *Muribaculaceae, Faecalibaculum, Romboutsia, Lactobacillus, Turicibacter*, and *A2* showed differences (*P*<0.05), and *Faecalibaculum* and *Romboutsia* were more abundant in HOB (Table 6), suggesting that *Faecalibaculum, Lactobacillus*, and *A2* were predominant in the obese groups, while *Romboutsia* and *Turicibacter* were predominant in hypoxia groups.

**Table 6:** Top 10 genus level gut microbiota difference.

**Genus level**	**Relevant abundance**				**Significant**			
	**LN**	**LOB**	**HN**	**HOB**	**LOB vs LN**	**HOB vs HN**	**HOB vs LOB**	**HN vs LN**
Muribaculaceae	0.350 ± 0.101	0.103 ± 0.066	0.348 ± 0.083	0.082 ± 0.026	0.001	0.001	0.421	0.964
Faecalibaculum	0.061 ± 0.159	0.282 ± 0.130	0.047 ± 0.147	0.141 ± 0.089	0.001	0.004	0.015	0.073
Dubosiella	0.229 ± 0.149	0.046 ± 0.145	0.090 ± 0.054	0.061 ± 0.083	0.005	0.337	0.742	0.022
Romboutsia	0.016 ± 0.012	0.023 ± 0.011	0.041 ± 0.023	0.210 ± 0.119	0.216	0.001	0.001	0.023
Lactobacillus	0.063 ± 0.026	0.164 ± 0.105	0.066 ± 0.021	0.031 ± 0.017	0.005	0.002	0.001	0.804
Helicobacter	0.009 ± 0.007	0.018 ± 0.012	0.020 ± 0.039	0.010 ± 0.014	0.285	0.590	0.281	0.713
Turicibacter	0.027 ± 0.013	0.023 ± 0.012	0.062 ± 0.030	0.011 ± 0.007	0.562	0.001	0.012	0.006
Bacteroides	0.023 ± 0.012	0.022 ± 0.013	0.035 ± 0.028	0.030 ± 0.025	0.846	0.662	0.439	0.271
A2	0.008 ± 0.003	0.024 ± 0.028	0.008 ± 0.002	0.031 ± 0.019	0.018	0.001	0.489	0.913
Erysipelatoclostridium	0.002 ± 0.001	0.004 ± 0.004	0.005 ± 0.002	0.022 ± 0.030	0.096	0.013	0.041	0.075

### Metabolic profiles of four groups of fecal metabolites

**Figure 3:**
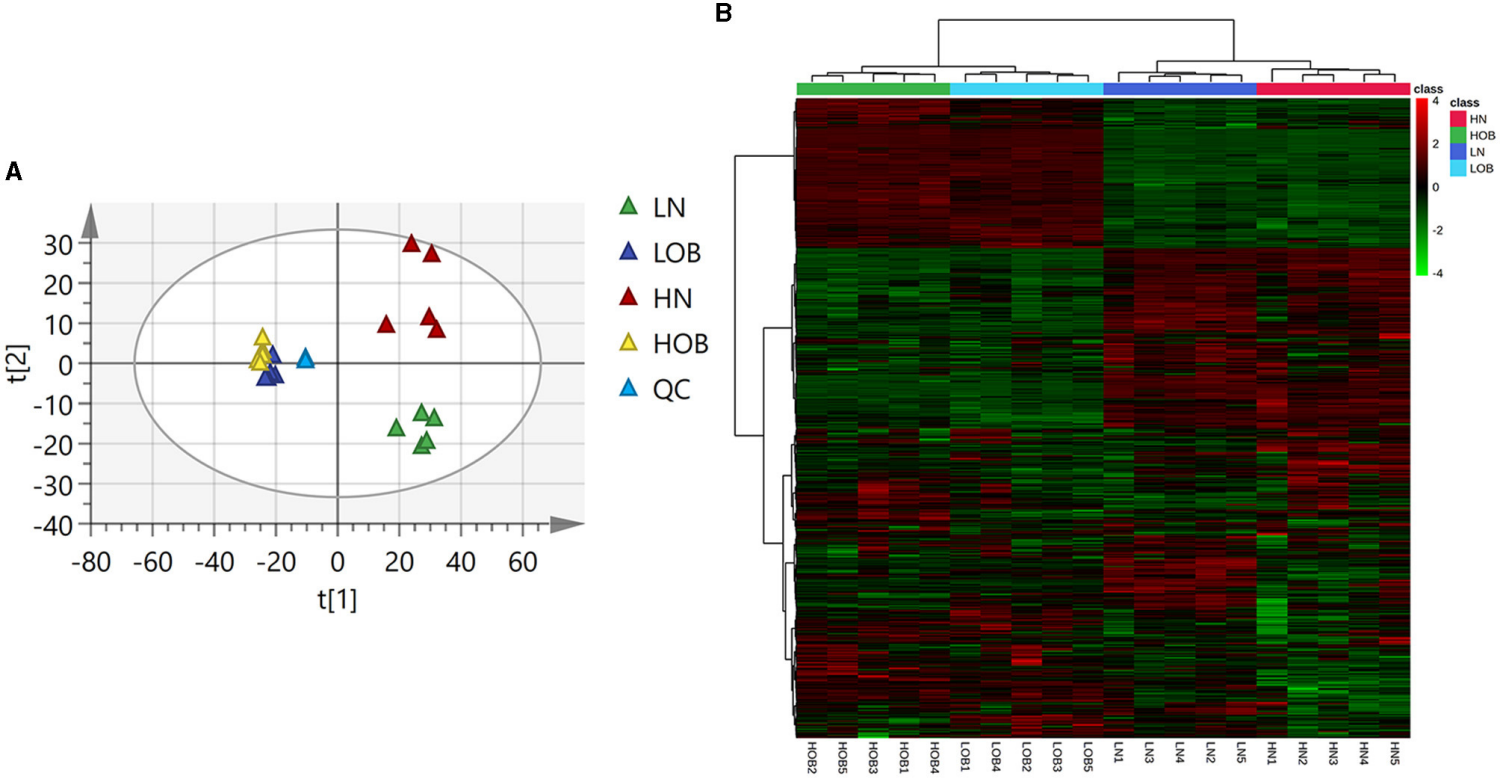
Metabolic profiles of four groups of fecal metabolites **(A)** PCA map. The distance of each coordinate point represents the degree of aggregation and dispersion between samples. **(B)** Clustering heatmap. **(A)** Heatmap provides intuitive visualization of a data table. Each colored cell on the map corresponds to a concentration value in your data table, with samples in rows and features/compounds in columns.

The PCA and clustering heatmap illustrate the differences between the sample distributions. The PCA map shows that the metabolites are clustered within groups and separated between groups (Figure 3A), and the clustering heatmap can roughly indicate that metabolites of the same body weight have similar expression patterns (Figure 3B).

### Differences in fecal metabolites among the four groups

**Figure 4:**
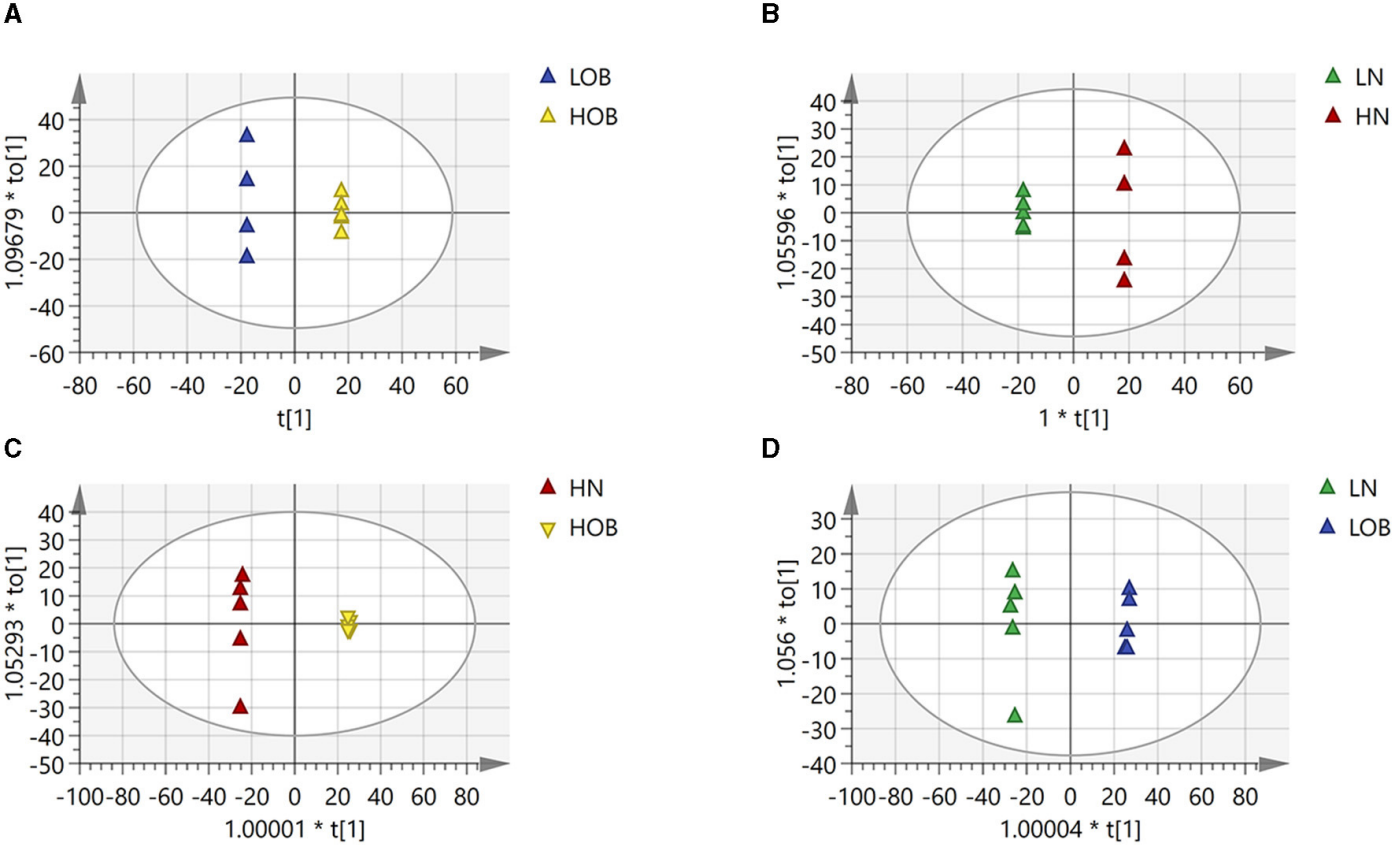
Differences in fecal metabolites between the groups (OPLADA map). **(A)** HOB vs. LOB group. **(B)** HN vs. LN group. **(C)** HOB vs. HN group. **(D)** LOB vs. LN group. The first prediction of X-axis is mainly the decomposition degree, and the first orthogonality of y-axis is the decomposition degree.

**Figure 5:**
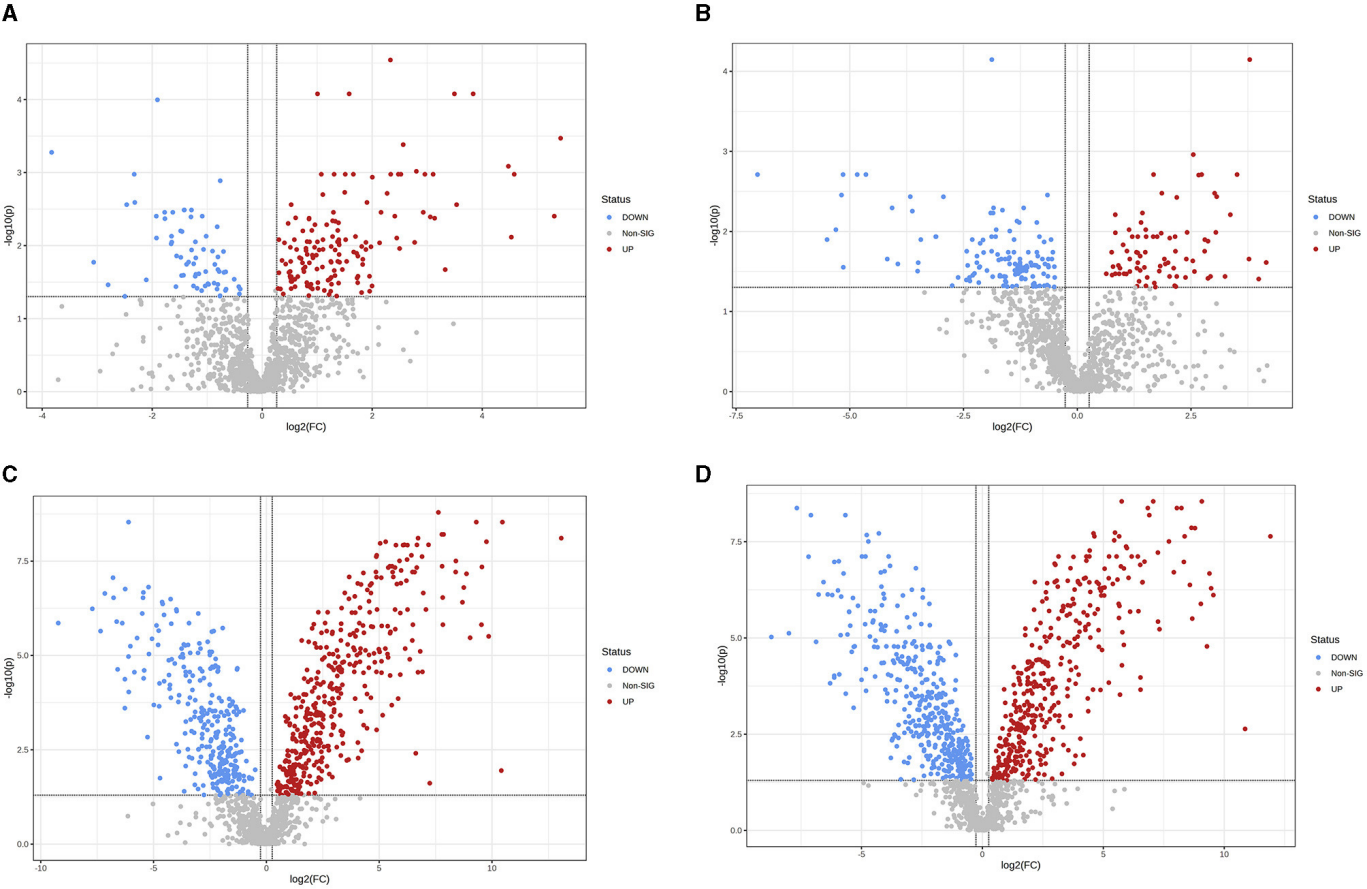
Differences in fecal metabolites between the groups (Volcano plot). **(A)** HOB vs. LOB. **(B)** HN vs. LN. **(C)** HOB vs. HN. **(D)** LOB vs. LN. Volcano plot combines results from Fold Change (FC) analysis and *T*-tests into one single graph, which intuitively select significant features based on either biological significance, statistical significance. Red dots indicate up-regulated metabolites, blue dots are vice versa.

**Figure 6:**
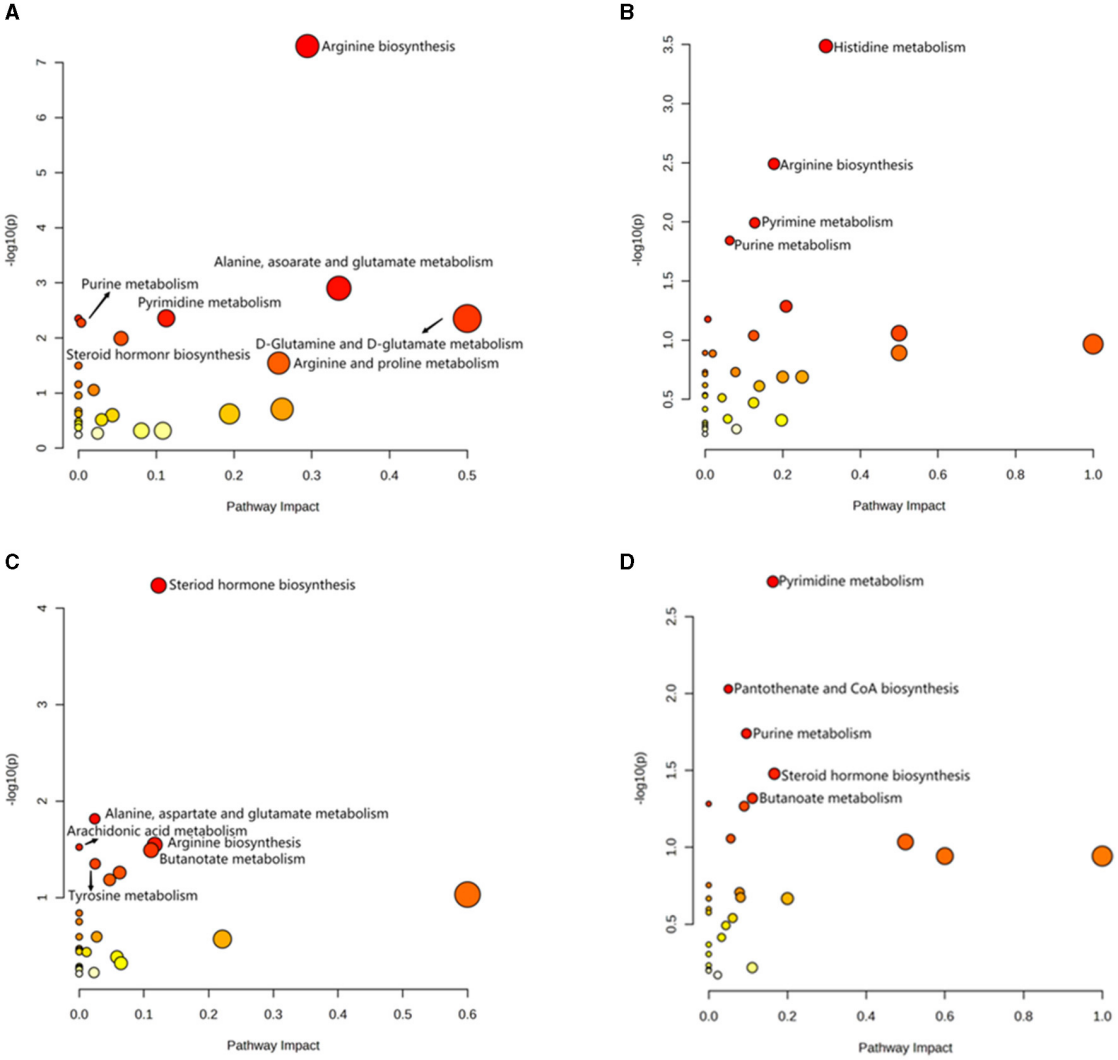
Analysis of KEGG pathway related to differential metabolites. **(A)** HOB vs. LOB. **(B)** HN vs. LN. **(C)** HOB vs. HN. **(D)** LOB vs. LN.

OPLS-DA model validation (Figure 4) revealed significant differences between the distribution of samples, suggesting a significant difference of fecal metabolites between the two groups, The model parameters are shown in Table 7, suggesting that the model was reliable. For the same body weight groups, i n HOB vs. LOB, a total of 736 different metabolites (*P* < 0.05, VIP > 1) were detected (Figure 5A), while 64 differential metabolites were successfully annotated, the majority of which were dominated by lipid compounds. KEGG pathway enrichment analysis of a total of 64 differential metabolites revealed that 60 differential metabolites were involved in pathway enrichment, involving a total of 26 metabolic pathways, of which seven pathways were significantly enriched (*P* < 0.05), namely arginine biosynthesis (*P* = 0.000), alanine, aspartate and glutamate metabolism (*P* = 0.001), pyrimidine metabolism (*P* = 0.004), D-Glutamine and D-glutamate metabolism (*P* = 0.004), purine metabolism (*P* = 0.005), steroid hormone biosynthesis (*P* = 0.010), and arginine and proline metabolism (*P* = 0.030) (Figure 6A). The enriched differential metabolites are shown in Table 8. In HN vs LN, a total of 737 differential metabolites were detected (*P* < 0.05, VIP > 1) (Figure 5B); 209 differential metabolites were successfully annotated. KEGG enrichment pathway analysis of a total of 209 differential metabolites revealed that 61 differential metabolites were involved in pathway enrichment, involving a total of 26 metabolic pathways, of which seven pathways were significantly enriched (*P* < 0.05), involving a total of 34 metabolic pathways, of which four pathways were significantly enriched (*P* < 0.05), namely histinide metabolism (*P* = 0.000), arginine metabolism (*P* = 0.002), primine metabolism (*P* = 0.023), and purine metabolism (*P* = 0.034) (Figure 6B); the enriched differential metabolites are shown in Table 9.

**Table 7:** OPLS-DA model parameters.

**Groups**	**A**	***N***	**R2X**	**R2Y**	**Q2**
HN-LN	1+4+0	10	0.747	0.997	0.993
HOB-LOB	1+5+0	10	0.816	1	0.945
LN-LOB	1+1+0	10	0.701	1	0.982
HN-HOB	1+1+0	10	0.688	0.998	0.963

A, the principal component score; R2X, the explanatory rate of the model to the X variable; R2Y, the explanatory rate of the model for the Y variable; Q2, the model's predictive ability.

**Table 8:** Relative abundance of significant differential metabolites involved in KEGG pathway analysis (HOB vs. LOB).

**Name**	**Mode**	**m/z**	**RT/min**	**VIP**	***P***	**FDR**	**FC**	**Trend**
L-Glutamine	pos	147.07687	1.312	1.416	0.003	0.025	0.433	↓
Uridine	neg	243.06261	2.385	1.514	0.000	0.001	22.261	↑
Fumaric acid	neg	115.00394	1.429	1.248	0.002	0.018	0.386	↓
Thymine	pos	127.05068	4.892	1.363	0.000	0.005	2.514	↑
L-Hydroxyproline	neg	307.11533	1.286	1.193	0.001	0.015	0.433	↓
Inosine	neg	267.0737	3.789	1.474	0.000	0.004	39.701	↑
Thymidine	neg	241.08347	4.884	1.178	0.006	0.038	2.015	↑
L-Ornithine	neg	131.08289	1.297	1.252	0.008	0.049	0.587	↓
2-Methoxyestrone	pos	301.17996	6.691	1.451	0.000	0.001	3.155	↑
Estriol	pos	289.17963	6.456	1.033	0.006	0.039	1.221	↑
cAMP	pos	330.05826	1.636	1.301	0.000	0.004	5.320	↑
L-Glutamic acid	pos	131.03448	1.321	1.449	0.000	0.004	0.470	↓
N-Acetylornithine	pos	175.10803	1.411	1.303	0.006	0.039	0.634	↓
Adenosine	pos	268.10471	3.155	1.279	0.000	0.003	11.592	↑
Cortisol	pos	363.21686	5.663	1.232	0.001	0.009	2.005	↑
Desoxycortone	pos	331.22726	6.329	1.256	0.000	0.008	2.150	↑
L-Argininosuccinate	neg	289.1156	1.302	1.249	0.006	0.039	0.566	↓
Guanosine	pos	284.09943	3.761	1.474	0.000	0.000	42.993	↑
Adrenosterone	pos	301.1799	6.179	1.414	0.001	0.015	1.897	↑

**Table 9:** Relative abundance of significant differential metabolites involved in KEGG pathway analysis (HN vs. LN).

**Name**	**Mode**	**m/z**	**RT/min**	**VIP**	***P***	**FDR**	**FC**	**Trend**
Methylimidazoleacetic acid	pos	1.369	141.0661	1.410	0.004	0.034	1.786	↑
Urocanic acid	pos	1.909	139.05069	1.498	0.000	0.006	1.784	↑
dCMP	neg	1.900	306.04962	1.289	0.000	0.012	0.116	↓
Creatine	pos	1.364	132.07698	1.166	0.006	0.039	15.866	↑
Histamine	pos	1.134	112.08722	1.036	0.006	0.040	0.185	↓
allantoate	pos	1.399	389.20242	1.355	0.008	0.049	0.511	↓
Uracil	pos	1.828	113.03475	1.396	0.004	0.034	0.600	↓
L-Glutamic acid	pos	1.321	131.03448	1.274	0.002	0.026	0.641	↓
N-Acetylornithine	pos	1.411	175.10803	1.358	0.003	0.031	0.404	↓
Cytidine	pos	1.727	244.09276	1.173	0.000	0.012	2.311	↑
Adenosine	pos	3.155	268.10471	1.431	0.005	0.038	2.902	↑
Ornithine	pos	1.144	133.09763	1.497	0.007	0.048	0.325	↓

For the same hypoxia level groups, in HOB vs. HN, a total of 705 differential metabolites (*P* < 0.05, VIP > 1) were detected (Figure 5C), while 121 differential metabolites were successfully annotated. The KEGG enrichment pathway analysis of a total of 121 differential metabolites revealed that 78 differential metabolites were involved in pathway enrichment, involving a total of 26 metabolic pathways, of which six pathways were significantly enriched (*P* < 0.05), namely steroid hoemone biosynthesis (*P* = 0.000), alainine, aspartate and glutamate metabolism (*P* = 0.015), arginine biosynthesis (*P* = 0.029), arachidonic acid metabolism (*P* = 0.030), butanoate metabolism (*P* = 0.032), and tyrosine metabolism (*P* = 0.044) (Figure 6C), the enriched differential metabolites are listed in Table 10. In LOB vs. LN, a total of 785 differential metabolites (*P* < 0.05, VIP > 1) were detected (Figure 5D); 143 differential metabolites were successfully annotated. The KEGG enrichment pathway analysis of a total of 143 differential metabolites revealed that 74 differential metabolites were involved in pathway enrichment, involving a total of 19 metabolic pathways, of which five pathways were significantly enriched (*P* < 0.05): pyrimidine metabolism (*P* = 0.002), pantothenate and CoA biosynthesis (*P* = 0.009), purine metabolism (*P* = 0.018), steroid hormone biosynthesis (*P* = 0.033), butanoate metabolism (*P* = 0.048), and tyrosine metabolism (*P* = 0.044) (Figure 6D). The enriched differential metabolites are shown in Table 11. These results suggest that hypoxia mainly affects nucleotide and amino acid metabolism, whereas obesity affects lipid and amino acid metabolism.

**Table 10:** Relative abundance of significant differential metabolites involved in KEGG pathway analysis (HOB VS HN).

**Name**	**Mode**	**m/z**	**RT/min**	**VIP**	***P***	**FDR**	**FC**	**Trend**
Prostaglandin I2	pos	353.23062	11.912	1.216	0.000	0.000	76.406	↑
Estradiol	pos	273.18478	6.407	1.257	0.000	0.000	9.954	↑
Fumaric acid	neg	115.00394	1.429	1.070	0.000	0.000	0.037	↓
Etiocholanolone	pos	273.22147	9.228	1.265	0.000	0.000	0.196	↓
Pregnenolone	neg	315.23352	9.836	1.079	0.000	0.000	0.027	↓
Estriol	pos	289.17963	6.456	1.287	0.000	0.000	32.960	↑
Succinic acid	neg	117.01951	2.692	1.032	0.001	0.002	0.063	↓
acetoacetate	pos	103.03941	6.634	1.277	0.000	0.000	231.080	↑
2,5-Dihydroxybenzaldehyde	neg	137.02498	5.372	1.143	0.004	0.009	0.380	↓
16(R)-HETE	pos	343.22513	8.25	1.275	0.000	0.000	0.198	↓
Desoxycortone	pos	331.22726	6.329	1.093	0.000	0.000	10.312	↑
Prostaglandin F2α	pos	337.23798	6.638	1.263	0.000	0.000	44.775	↑
L-Argininosuccinate	neg	289.1156	1.302	1.076	0.024	0.046	0.307	↓
Adrenosterone	pos	301.1799	6.179	1.283	0.000	0.000	11.118	↑

**Table 11:** Relative abundance of significant differential metabolites involved in KEGG pathway analysis (LOB vs. LN).

**Name**	**Mode**	**m/z**	**RT/min**	**VIP**	***P***	**FDR**	**FC**	**Trend**
2′-Deoxyadenosine 5′-monophosphate	neg	330.06134	2.111	1.150	0.000	0.000	0.046	↓
dCMP	neg	306.04962	1.9	1.061	0.000	0.000	0.056	↓
5′-Adenylic acid	pos	348.06906	1.373	1.221	0.003	0.006	0.216	↓
Dehydroepiandrosterone	pos	289.21661	6.269	1.045	0.000	0.000	3.414	↑
Adenosine 5′-monophosphate	neg	346.05624	3.327	1.091	0.000	0.000	0.035	↓
Xanthine	pos	153.04117	2.211	1.226	0.014	0.026	0.602	↓
Cytidine-5′-monophosphate	pos	324.05881	5.453	1.253	0.000	0.000	8.871	↑
Pregnenolone	neg	315.23352	9.836	1.028	0.000	0.000	0.081	↓
Estriol	pos	289.17963	6.456	1.257	0.000	0.000	22.049	↑
2′-Deoxyadenosine	pos	252.10947	4.666	1.075	0.000	0.001	0.137	↓
Succinic acid	neg	117.01951	2.692	1.173	0.001	0.002	0.147	↓
Uric acid	pos	113.03475	1.828	1.188	0.020	0.035	6.456	↑
Pantetheine	pos	279.13684	1.29	1.068	0.000	0.000	6.656	↑
acetoacetate	pos	103.03941	6.634	1.248	0.000	0.000	119.600	↑
Adenosine	pos	268.10471	3.155	1.084	0.000	0.001	0.217	↓
Orotic acid	neg	155.01024	1.812	1.135	0.006	0.011	0.225	↓
Deoxycytidine	pos	228.09785	1.414	1.147	0.000	0.001	0.347	↓
Desoxycortone	pos	331.22726	6.329	1.210	0.000	0.000	5.582	↑
Pantothenic acid	pos	220.11832	5.075	1.234	0.001	0.002	0.575	↓

### Correlation analysis of fecal metabolites with gut microbiota

**Figure 7:**
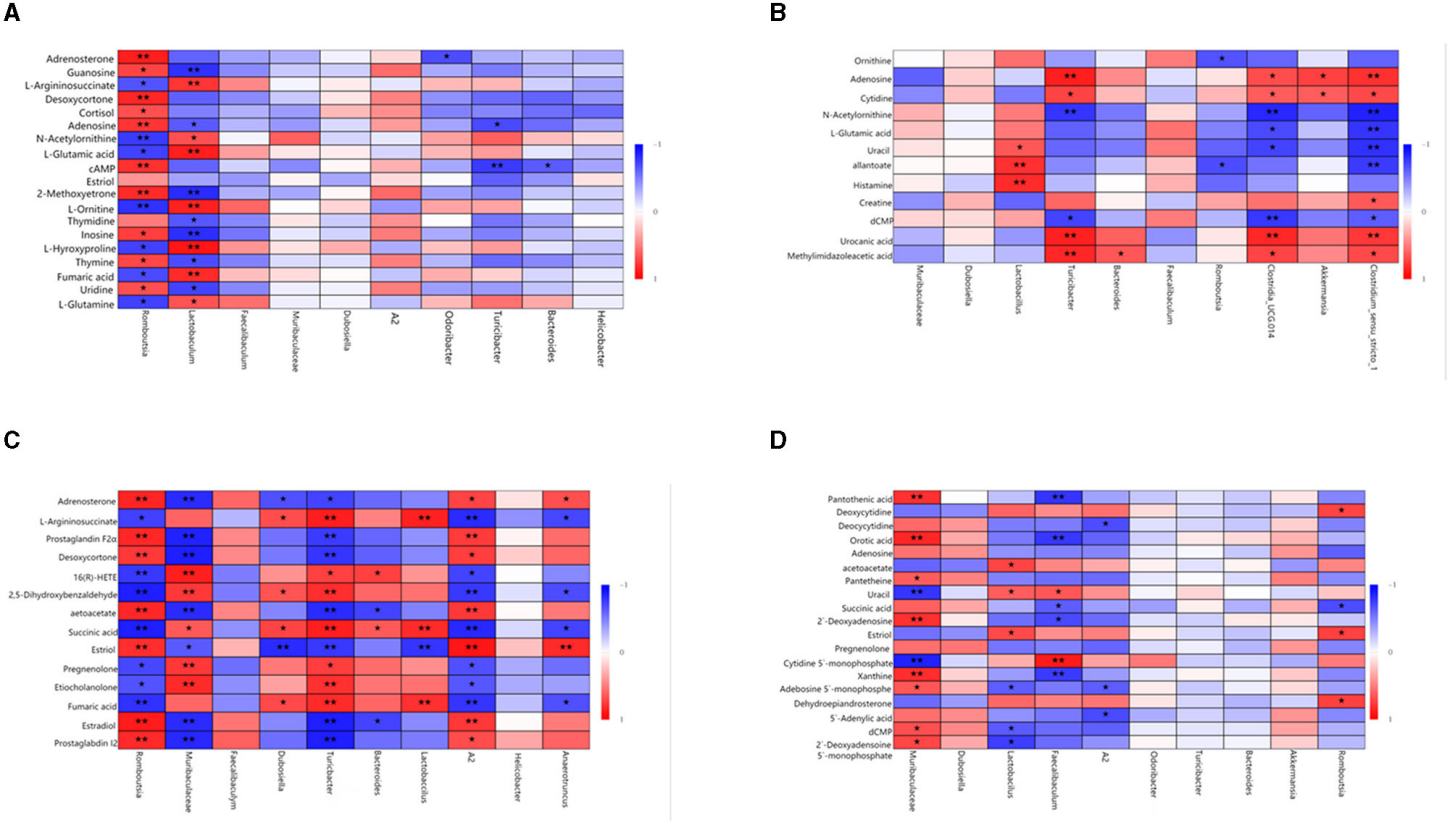
Correlation analysis of gut microbiota and fecal metabolites. **(A)** HOB vs. LOB. **(B)** HN vs. LN. **(C)** HOB vs. HN. **(D)** LOB vs. LN. The red line represents a significant positive correlation, while the blue line represents a significant negative correlation, ^*^P < 0.05; ^**^P < 0.01.

Spearman's correlation was used to investigate the relationship between gut microbiota and metabolites. The relationship between the most differentially expressed metabolites and the top 10 genera was analyzed in mice (Figure 7). In the same body weight groups, in HOB vs. LOB, we found that the abundance of *Romboutsia* was positively correlated with the fecal lipid compounds adrenosterone, desoxycortone, 2-methoxyestrone, and cortisol (*P* < 0.05), and with that of fecal nucleotide compounds guanosine, cAMP, adenosine, uridine, and inosin (*P* < 0.05), but negatively correlated with organic acids L-glutamine, fumaric acid, L-hydroxyproline, L-ornithine, L-glutamic acid, N-acetylornithine, and L-argininosuccinate (*P* < 0.05). *Lactobaculum* was positively correlated with the organic acids L-glutamine, fumaric acid, L-hydroxyproline, L-ornithine, L-glutamic acid, N-acetylornithine, and L-argininosuccinate (*P* < 0.05), but there was a negative correlation with nucleotides guanosine, thymidine, adenosine, uridine, and inosine (*P* < 0.05). This is the opposite of *Romboutsia* in relation to metabolites (Figure 7A). In HN vs. LN, *Turicibacter* was positively correlated with the nucleotide compounds adenosine, urocanic acid, and cytidine (*P* < 0.05) and *Romboutsia* was negatively correlated with the organic acid compounds allantoate and ornithine (*P* < 0.05). These results suggested that the microbial *Romboutsia* characterized by hypoxia showed a negative correlation with organic acid compounds and a positive correlation with nucleotide and lipid compounds (*P* < 0.05) (Figure 7B).

In the same oxygen level groups, in HOB vs. HN, *Romboutsia* was positively correlated with the fecal lipid compounds prostaglandin I2, estradiol, estriol, desoxycortone, prostaglandin F2α, and adrenosteron (*P* < 0.05), but negatively correlated with the organic acid L-argininosuccinate, succinic acid, and fumaric acid (*P* < 0.05). *Muribaculaceae* was negatively correlated with the fecal lipid compound prostaglandin I2, estradiol, estriol, desoxycortone, prostaglandin F2α, and adrenosteron (*P* < 0.05), but positively correlated with the organic acid succinic acid. *Lactobacillus* was positively correlated with fumaric acid, succinic acid, and L-argininosuccinat (*P* < 0.05). *A2* was positively correlated with prostaglandin I2, estradiol, estriol, desoxycortone, prostaglandin F2α, and adrenosteron (*P* < 0.05), but negatively correlated with fumaric acid, succinic acid, and L-argininosuccinat (*P* < 0.05) (Figure 7C). In LOB vs. LN, *Muribaculaceae* was positively correlated with nucleotides 2′-deoxyadenosine 5′-monophosphate, dCMP, adenosine 5′-monophosphate, xanthine, and 2′-deoxyadenosin (*P* < 0.05); *Lactobacillus* was negatively correlated with 2′-deoxyadenosine 5′-monophosphate, dCMP, and adenosine 5′-monophosphat (*P* < 0.05). *Faecalibaculum* was negatively correlated with orotic acid, succinic acid, and pantothenic acid (*P* < 0.05), but positively correlated with uracil and cytidine-5′-monophosphat (*P* < 0.05). It was positively correlated with the fecal lipid compound estriol (*P* < 0.05) and with correlated with acetoacetat (*P* < 0.05). *A2* was negatively correlated with 5′-adenylic acid, adenosine 5′-monophosphate, and deoxycytidin (*P* < 0.05) (Figure 7D). These results suggested that gut microbiota in the hypoxic group were positively correlated with lipid and nucleotide compounds and negatively correlated with organic acid compounds.

## Discussion

In this study, we compared the gut microbiota and feal metabolites of obese and normal weight mice with different oxygen levels using 16S rRNA sequencing and metabolomics. We found that (1) the diversity of the hypoxic and obese gut microbiota was reduced compared to the control groups. (2) Hypoxia and obesity lead to disturbances in gut metabolism. (3) Gut microbiota and fecal metabolism are closely related. Identifying the characteristic bacteria and metabolites of obesity in different environments will help us to target interventions for obesity.

Studies have shown that low-grade inflammation is a hallmark of obesity, characterized by changes in the composition of the gut microbiota and its metabolites, which are transferred from the gut across the disrupted intestinal barrier and affect various metabolic organs, such as the liver and adipose tissue, thereby promoting metabolic inflammation (Tilg et al., 2020). Available evidence on the correlation between metabolomics and obesity metabolite profiles suggests that it is mainly related to amino acid and lipid metabolites (Payab et al., 2022). In this study, we found that in the hypoxic obesity group (HOB vs. HN), changes were mainly caused in *Romboutsia, Muribaculaceae, Lactobacillus* and *A2*, and in the metabolism of amino acids and lipid compounds. Among them, *Romboutsia* showed a positive correlation with lipid compounds Prostaglandin I2 (FC = 76.406), Estriol (FC = 32.960), Prostaglandin F2α (FC = 44.775), Adrenosterone (FC = 11.118), and Desoxycortone (FC=10.312). Studies have shown that *Romboutsia, Erysipelatoclostridium*, and *Enterobacteriaceae* are positively associated with obesity and intestinal inflammation (Nagayama et al., 2020; Yu et al., 2020; Song et al., 2021), and suggested that the *Romboutsia-*driven microbiome, characterized by low bacterial diversity and high primary bile acids, is associated with fat-driven obesity (Therdtatha et al., 2021). Thus, we hypothesized that hypoxic obesity is associated with *Romboutsia-*driven ecological dysregulation of the gut microbiome. Among these metabolic compounds, Prostaglandin I2 and Prostaglandin F2α, as bioactive lipid mediators, are associated with inflammation (Idborg and Pawelzik, 2022), and 8-iso-PGF2α are associated with obesity (Furukawa et al., 2004), and both compounds are involved in the metabolism of arachidonic acid, which can be metabolized to leukotriene A4 (LTA4) and lipoxin (LXs), and further generate other types of leukotriene (LT)(Guriec et al., 2014). LT can activate leukocytes and promote inflammation (Bertin et al., 2014). Studies have shown that steroid hormones are associated with metabolic diseases in women (e.g., obesity and gestational diabetes), whereas estradiol increases energy expenditure by increasing thermogenesis and lipolysis of BAT (Vigil et al., 2022). The metabolism of these lipid compounds may also be associated with hypoxic factors. One study has suggested that hypoxia is also an important trigger for fatty acid oxidation (Hikita et al., 2007). Amino acids are not only essential nutrients and energy sources but are also involved in many biochemical processes such as purine biosynthesis and uric acid production. The main organ involved in amino acid metabolism is the liver, which plays an important role in maintaining amino acid homeostasis. Abnormal amino acid metabolism results in dysregulation of fatty acid, protein, and urea synthesis, energy metabolism, protein hydrolysis, and cell signaling (Newsholme et al., 2003). Previous studies found that compounds such as alanine, glutamate, proline, succinate, tyrosine, and BCAA were found to be higher in obese participants (Butte et al., 2015), however, our study found a downregulation trend in hypoxic obese mice. Many organic acids are involved in the TCA cycle and their metabolism is disturbed in obese mice, affecting energy metabolism, but the exact mechanism is not clear. In the LOB vs. LN group, a higher abundance of *Faecalibaculum, Lactobacillus*, and *A2* was found in the normoxic group. *Lactobacillus*, which is particularly abundant in obese mice, can regulate body weight by hydrolyzing indigestible polysaccharides into easily absorbed monosaccharides, thereby activating lipoprotein lipase, and it regulates DNA methylation levels at the host miR-378a promoter by increasing acetate and butyrate in SCFAs, thereby preventing the development of obesity and glucose intolerance (Du et al., 2021). *Faecaalibaculum* is mainly present in obese individuals and can maintain intestinal homeostasis by producing butyrate, inhibiting NF-KB, and upregulating PPAR-γ to suppress the onset of inflammation (Gomes et al., 2018). In this study, we found that *Faecalibaculum* showed a positive correlation with lipid and nucleotide compounds. In a high-fat diet-induced gut microbiota sequencing of obese mice, *Colidextribacter* and *Faecalibacterium* were found to be indicators of obesity and their abundance was positively correlated with obesity. Therefore, we speculate that the occurrence of obesity in the normoxic group may be related to the disruption of the gut microbiota induced by *Faecalibacterium* (Yu et al., 2022). Pyrimidine and purine nucleotide metabolism disorders predominate, suggesting that under conditions of cellular damage (inflammation, hypoxia, acute injury), ATP is rapidly released from cells into the extracellular compartment, causing a rapid increase in extracellular levels of ATP, ADP, or AMP, and adenosine produced by hydrolysis (Fredholm, 2007). Some studies have found that obesity increases the breakdown of adenine nucleotides in adipose tissue (Tsushima et al., 2013), and xanthine is one of the representative metabolites. Xanthine is a common host-microbe metabolite found in human tissues, body fluids, and intestinal flora, and can be converted to uric acid in mice. Both xanthine and hypoxanthine are precursors of oxidized purines and uric acid, are oxidized by xanthine oxidoreductase in purine catabolism, and play an important role in ATP generation (Nishino and Okamoto, 2015). Studies have shown that uric acid is the final oxidation product of purine nucleotide catabolism in humans, that serum uric acid levels are based on the balance between the absorption, production, and excretion of purines and purine nucleotides, and that obesity is often associated with hyperuricemia (Tsushima et al., 2013). Elevated serum uric acid is strongly associated with visceral fat accumulation and several metabolic diseases (Tamba et al., 2008; Kim et al., 2012). In this study, uric acid levels showed an upregulation trend in the obese groups (FC=6.456), which is consistent with the above conclusions.

In the same body weight groups (HOB vs. LOB, HN vs. LN), the main dominant bacterium was *Romboutsia*, which was mainly associated with the metabolism of nucleotide and amino acid compounds. The analysis of the effect of altitude on Sanhe heifers' gut microbiota and metabolism found that *Romboutsia* was associated with the metabolism of purine pyrimidines and amino acids (Zhang et al., 2023). Another study found that some potential probiotics, including *Christensenellaceae_R-7_group, Ruminococcus_1, Romboutsia, Alloprevotella, E. coprostanoligenes*, and *Clostridium*, were enriched in the rumen of high-altitude yaks. Shifts in the rumen microbiomes were caused by a high-altitude environment characterized by cold temperatures, hypoxia, and the production of high-fiber herbage. Moreover, rumen microbial diversity and herbage fermenting ability of yaks increased with elevation; therefore, high-altitude yaks should be considered to have microbiota adaptation to partially meet the higher energy requirements needed for survival in the harsh cold and hypoxic environment (Fan et al., 2020). In an *in vivo* study, it was found that *Romboutsia* was significantly associated with bile acids, triglycerides, amino acids and derivatives, and organic acids and derivatives in the standard diet groups, whereas triglycerides and free fatty acids were significantly associated in the high-fat diet groups (Yin et al., 2023). A large body of research suggests that hypoxic environments can alter lipid metabolism in mice and humans (Famulla et al., 2012; Siques et al., 2020; Morin et al., 2021). It may be that the hypoxic environment at high altitude promotes fat mobilization.

This study only analyzed changes in metabolites in mouse feces, and so cannot fully elucidate systemic metabolic changes in mice. At the same time, during the hypoxic intervention, the effect of oxygen concentration on mice at different time points cannot be displayed dynamically without dividing the time periods. Due to the small sample size, the construction of models for predicting the onset of obesity by metabolites is limited. Therefore, based on this study, further sample expansion and targeted metabolomics research with multiple time points and tissues can be carried out in subsequent studies, providing new ideas for the potential molecular mechanisms of preventing or treating obesity in high-altitude areas.

## Conclusions

In conclusion, 16S rRNA gene sequencing and untargeted metabolomics revealed characteristic changes in fecal metabolites and gut microbiota in obese and hypoxic groups of mice. In the same body weight groups, we found that the dominant flora was associated with the metabolism of nucleotide and amino acid compounds. In the same oxygen concentration groups, we found that the dominant bacteria were associated with lipids and amino acids.

## Data availability statement

The datasets presented in this study can be found in online repositories. The names of the repository/repositories and accession number(s) are: NCBI SRA (accession: PRJNA1007439).

## Ethics statement

The animal study was approved by the Medical Science Research Ethics Committee of Qinghai University (No. 2021-06, approved 21 June 2021). The study was conducted in accordance with the local legislation and institutional requirements.

## Author contributions

AA and WD conceived and supervised the study. WD, AA, XY, YM, and ZW collected and processed samples. XW provided financial support. AA, XY, and SL analyzed data and drafted the manuscript. All authors contributed to the article and approved the submitted version.
